# Cumulative incidence of motor and cognitive features in the amyotrophic lateral sclerosis—frontotemporal degeneration spectrum

**DOI:** 10.1093/braincomms/fcaf405

**Published:** 2025-10-15

**Authors:** Barbara E Spencer, Sharon X Xie, Daniel T Ohm, Lauren Elman, Colin C Quinn, Defne A Amado, Michael Baer, Edward B Lee, Vivianna M Van Deerlin, Laynie Dratch, Lauren Massimo, David J Irwin, Corey T McMillan

**Affiliations:** Department of Neurology, University of Pennsylvania, Philadelphia, PA 19104, USA; Department of Biostatistics, Epidemiology and Informatics, University of Pennsylvania, Philadelphia, PA 19104, USA; Department of Neurology, University of Pennsylvania, Philadelphia, PA 19104, USA; Department of Neurology, University of Pennsylvania, Philadelphia, PA 19104, USA; Department of Neurology, University of Pennsylvania, Philadelphia, PA 19104, USA; Department of Neurology, University of Pennsylvania, Philadelphia, PA 19104, USA; Department of Neurology, University of Pennsylvania, Philadelphia, PA 19104, USA; Department of Pathology and Laboratory Medicine, University of Pennsylvania, Philadelphia, PA 19104, USA; Department of Pathology and Laboratory Medicine, University of Pennsylvania, Philadelphia, PA 19104, USA; Department of Neurology, University of Pennsylvania, Philadelphia, PA 19104, USA; Department of Neurology, University of Pennsylvania, Philadelphia, PA 19104, USA; Department of Neurology, University of Pennsylvania, Philadelphia, PA 19104, USA; Department of Neurology, University of Pennsylvania, Philadelphia, PA 19104, USA

**Keywords:** epidemiology, amyotrophic lateral sclerosis, frontotemporal degeneration, dementia

## Abstract

In frontotemporal degeneration and amyotrophic lateral sclerosis, subsequent motor or cognitive-behavioural features, respectively, are associated with shorter survival. However, factors influencing subsequent feature development remain largely unexplored. In this study, we examined whether the presence of a *C9orf72* expansion or the initial clinical syndrome was associated with increased risk of subsequent feature development in individuals with amyotrophic lateral sclerosis and frontotemporal degeneration. We performed a retrospective evaluation of the entire disease course of individuals with an initial clinical syndrome of amyotrophic lateral sclerosis or frontotemporal degeneration who had neuropathological confirmation of TDP-43 proteinopathy at autopsy or a *C9orf72* hexanucleotide repeat expansion. We examined the odds and hazard of subsequent feature development and assessed whether each was modified by the presence of a *C9orf72* expansion or initial clinical syndrome. At autopsy, we evaluated the association between TDP-43 pathology burden in characteristic brain regions and features across this disease spectrum. For individuals with amyotrophic lateral sclerosis (*n* = 168) and frontotemporal degeneration (*n* = 73), binary logistic regression revealed increased odds (odds ratio = 3.49 [95% confidence interval 1.64–7.80], *P* = 0.002) and Cox proportional hazard analyses revealed an increased hazard (hazard ratio = 3.78 [95% confidence interval 1.86–7.65], *P* < 0.001) for developing subsequent features in those with a *C9orf72* expansion compared to those without. Beyond *C9orf72* expansion status, binary logistic regression revealed decreased odds (odds ratio = 0.25 [95% confidence interval 0.12–0.53], *P* < 0.001) and Cox proportional hazard analyses revealed a decreased hazard (hazard ratio = 0.48 [95% confidence interval 0.25–0.95], *P* = 0.03) for developing subsequent features in those with an initial amyotrophic lateral sclerosis clinical syndrome compared to those with an initial frontotemporal degeneration clinical syndrome. We observed a 94-month difference in the time after symptom onset of the initial clinical syndrome that a given person without a *C9orf72* expansion reached the highest probability of developing subsequent features (0.12 [95% CI 0.03–0.19], 113.00 months) and a person with a *C9orf72* expansion surpassed that probability (0.13 [95% CI 0.06–0.19], 19.00 months). The distribution of TDP-43 pathology across characteristic brain regions reflected both the initial clinical syndrome and subsequent features, with relatively preserved spinal cord only in frontotemporal degeneration cases without subsequent motor features (*P* < 0.0001) and relatively preserved neocortical regions only in amyotrophic lateral sclerosis cases without subsequent cognitive-behavioural features (*P* < 0.0001). These data highlight the need for clinician vigilance to detect the onset of subsequent motor and cognitive-behavioural features in patients carrying a *C9orf72* expansion, regardless of initial clinical syndrome. *C9orf72* clinical care can be enhanced through coordination between cognitive and neuromuscular clinics.

## Teaser

Spencer *et al.* demonstrated both the presence of a *C9orf72* expansion and the initial clinical syndrome modify risk of subsequent feature development in frontotemporal degeneration and amyotrophic lateral sclerosis, highlighting the need for clinician vigilance to detect the onset of subsequent motor and cognitive-behavioural features in this disease spectrum.

## Graphical Abstract

**Graphical Abstract:**
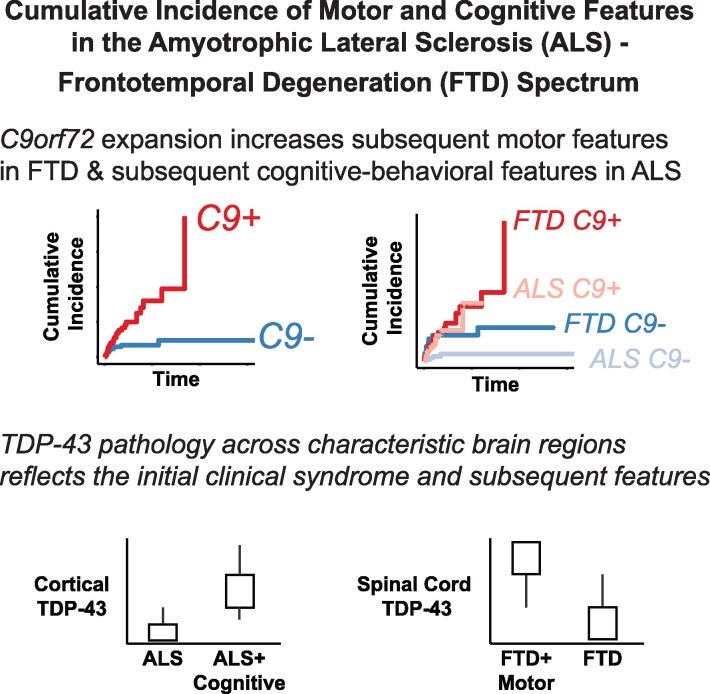

## Introduction

Frontotemporal degeneration (FTD) and amyotrophic lateral sclerosis (ALS) are multi-system disorders that occur along a spectrum of cognitive-behavioural and neuromuscular impairments, respectively.^1^ Pathologic, genetic, and clinical features support the existence of this clinicopathologic spectrum. First, TAR DNA-binding protein ∼43 kDa (TDP-43) inclusions are the pathological hallmark of ∼50% of FTD and the vast majority (>98%) of ALS cases.^2^ Second, hexanucleotide repeat expansions in *C9orf72*^3,4^ can cause FTD, ALS, or both, and account for ∼5% of simplex and one-third of familial FTD and ALS cases.^5,6^ Third, clinical overlap between FTD and ALS can occur, even in the absence of a *C9orf72* expansion, though estimates of the relative frequency of subsequent feature development vary widely.

In both FTD and ALS, the presence of subsequent motor or cognitive-behavioural features, respectively, is associated with shorter survival.^7-10^ Therefore, understanding the risk of subsequent features within FTD and ALS is critical, yet factors influencing the risk of subsequent feature development remain largely unexplored.

Previous studies estimate that ∼9–15% of individuals with ALS eventually develop frank FTD, though 30–50% develop some level of cognitive impairment.^1,11-15^ Recently, using appropriate normative data, we found that ∼15% of individuals with ALS have cognitive impairment.^16^ Likewise, ∼4–14% of individuals with FTD develop a subsequent motor impairment consistent with ALS,^17-20^ a number that may underestimate the incidence since latent evidence of neuromuscular impairment is rarely assessed. Estimates of subsequent motor or cognitive-behavioural feature development specifically in those with disease due to a *C9orf72* expansion vary widely (∼11–60%).^3,21-25^ It is difficult to compare estimates across studies that use different criteria to determine the presence of subsequent features. In several studies that directly compare persons with *C9orf72* expansions to those without *C9orf72* expansions, a higher frequency of subsequent feature development has been reported in individuals with *C9orf72* expansions (23–50%) compared to those without *C9orf72* expansions (4–12%).^26-28^ However, research limited to individuals with *C9orf72* expansions likely overestimates concomitant FTD and ALS.

A further major limitation of prior research evaluating subsequent feature development in FTD and ALS is the reliance on clinically rather than neuropathologically defined cases. Upper and/or lower motor neuron features are extraordinarily rare in FTD due to underlying tau pathology.^29,30^ Tau pathology accounts for nearly half of all FTD cases and is not easily clinically differentiated from FTD due to underlying TDP-43. Thus, subsequent ALS in FTD is likely underestimated when individuals without an underlying TDP-43 proteinopathy are included in risk models.

Recent evidence suggests that the risk of developing subsequent ALS in individuals with an initial presentation of FTD decreases with time from FTD symptom onset.^27^ However, we are unaware of studies evaluating the hazard of subsequent feature development over the disease course, which is an important consideration for patient prognostication. Further, the hazard of subsequent feature development stratified by initial clinical syndrome, FTD or ALS, has not yet been explored.

In this study, we examine whether the presence of a *C9orf72* expansion is associated with increased risk of subsequent feature development in individuals with ALS, excluding those with *SOD1*-related disease lacking TDP-43 pathology, and FTD, excluding those with non-TDP-43 proteinopathy at autopsy. We further ask whether the risk of subsequent feature development is modified by the initial clinical syndrome, FTD or ALS, beyond *C9orf72* expansion status. Using a targeted, retrospective evaluation of patients with a confirmed TDP-43 proteinopathy who were followed clinically from initial diagnosis until death, we are able to assess the presence or absence of subsequent feature development through the entire disease course. At autopsy, we evaluate the distribution of TDP-43 pathology in characteristic brain regions to evaluate the association between pathology and features across the FTD-ALS spectrum.

## Materials and methods

### Participants

We retrospectively evaluated data from 241 deceased individuals from the University of Pennsylvania Integrated Neurodegenerative Disease Database.^31,32^ Informed consent was obtained for all participants through a procedure approved by an Institutional Review Board convened at the University of Pennsylvania.

Inclusion was limited to individuals with:

1. An initial clinical syndrome consistent with ALS or FTD as evaluated by a board-certified neurologist with expertise in neuromuscular and/or cognitive disorders, and
2. Complete records for date of birth, date of symptom onset for the initial clinical syndrome, and date of death, and
3. Blood DNA or post-mortem frozen brain tissue screened for the presence or absence of a *C9orf72* expansion (see ‘Genetic analysis’ section), and
4. Neuropathological confirmation of a TDP-43 proteinopathy at autopsy (see ‘Neuropathological evaluation’ section) *OR* the presence of a *C9orf72* expansion, and
5. Chart review

Two qualified reviewers performed a retrospective chart review of all available electronic and paper health records for each individual. On average, individuals with ALS were seen clinically every 3 months, and those with FTD were seen clinically every 6 months. Each reviewer independently assessed each case and recorded the presence or absence of subsequent features during disease course and, if present, the date of onset of subsequent features. Discrepancies between reviewers were reconciled by a third reviewer, and Cohen's Kappa was used to measure inter-rater reliability. Subsequent features were defined as any clinical evidence of motor impairment in an individual with an initial clinical presentation of FTD or any clinical evidence of cognitive-behavioural impairment in an individual with an initial clinical presentation of ALS. Subsequent motor features included irregular or slowed gait, foot drop, dysphagia, dysarthria, fasciculations, limb weakness, and shortness of breath. Subsequent cognitive-behavioural features included personality change, disinhibition, apathy, compulsive or ritualistic behaviours, agitation, deficits in executive function, lack of empathy, and lack of insight. We did not consider the following as subsequent features: Parkinsonism such as rigidity, pseudobulbar features like inappropriate laughter or crying, or decades long behavioural features lacking clear evidence of progression. When month was not determinable the default of January was used, and when day was not determinable, the first of the month was used. Time to subsequent feature development was calculated as the difference in months between the date of symptom onset of the initial clinical syndrome and the date of onset of subsequent features.

### Genetic analysis

DNA was extracted from peripheral blood or frozen brain tissue following the manufacturer’s protocols [QuickGene DNA whole blood kit (Autogen) for blood, and QIAamp DNA Mini Kit (Qiagen) for brain tissue]. All individuals were tested for *C9orf72* hexanucleotide repeat expansions using a modified repeat-primed PCR as previously described. Cases were defined as having a pathogenic *C9orf72* expansion if >30 hexanucleotide repeats were identified. Individuals were further screened for pathogenic variants associated with FTD and/or ALS using exome/genome sequencing, and/or a custom targeted multi-neurodegenerative disease sequencing panel.^31^ The sequencing data were analysed using Geneticist Assistant software (Soft Genetics, State College, PA).

### Neuropathological evaluation

Detailed neuropathological assessments were performed using established and uniform methods of fixation, tissue processing, IHC with well-characterized antibodies, and current neuropathological criteria, as has been described in detail elsewhere.^31,33,34^ Briefly, 15 brain regions from one hemisphere, alternating right and left at random, are routinely sampled at autopsy, formalin-fixed, and processed for immunohistochemical staining using 1D3 (gift of Manuela Neumann and Elisabeth Kremmer) for phosphorylated TDP-43. Each brain region was semi-quantitatively scored for the burden of TDP-43 (0, absent; 0.5, rare; 1, mild; 2, moderate; 3, severe; Supplementary Fig. 1).

### Statistical analysis

We first assessed the effect of *C9orf72* expansion status (present or absent) and initial clinical syndrome (FTD or ALS) on the odds of developing subsequent features at any point from symptom onset of the initial clinical syndrome until death using binary logistic regression, controlling for age at initial clinical syndrome onset and sex. Odds ratio (OR) and confidence interval (CI) are reported. We next assessed the effect of *C9orf72* expansion status, alone and in combination with initial clinical syndrome on subsequent feature development using a time-to-event model.

The Kaplan–Meier method was used to estimate the cumulative incidence of subsequent feature development. An individual was considered at risk for subsequent feature development from the time of symptom onset of the initial clinical syndrome until death. We subsequently considered an individual at risk until either death, tracheostomy, or permanent assisted ventilation, consistent with the definition of survival in some clinical trials.^35^ Here, permanent assisted ventilation was defined as the date at which the individual reported using non-invasive positive pressure ventilation for more than 23 h per day.

Cox proportional hazard analyses, controlling for age at initial clinical syndrome onset and sex, were used to examine the influence of *C9orf72* expansion status, alone and in combination with initial clinical syndrome, on subsequent feature development. Hazard ratio (HR) and CI are reported. The proportional hazards assumption was tested for each Cox regression model fit.^36^

Finally, the distribution of TDP-43 pathology was evaluated across the FTD-ALS spectrum, using FDR-adjusted Kruskal–Wallis tests to evaluate the difference in TDP-43 burden in each characteristic brain region across individuals grouped by initial clinical syndrome and subsequent features, followed by FDR-adjusted pairwise Dunn’s tests to identify which groups were different. All statistical tests were two-sided. All analyses were done using R (version 4.2.3).

### Data availability

Diagnosis, genetic, and neuropathological data may be requested and upon approval of reasonable requests may be shared with individual investigators. Data requests can be completed through the Penn Neurodegenerative Data Sharing Committee webform: https://www.pennbindlab.com/data-sharing.

## Results

### Subsequent feature development

Seventy-three individuals with initial FTD (35 [48%] *C9orf72* expansion present) and 168 individuals with initial ALS (65 [39%] *C9orf72* expansion present) were included in the study. Demographic, clinical, and genetic characteristics for all individuals are summarized in Table 1. Of the 73 individuals with initial FTD, 22 (30%) developed subsequent motor features (Supplementary Table 1). Of the 168 individuals with initial ALS, 16 (10%) developed subsequent cognitive-behavioural features. Cohen's Kappa indicated very high agreement in the assessment of each case for the presence or absence of subsequent features (0.84). Of the 38 individuals who developed subsequent features, regardless of *C9orf72* status, 27 did so prior to the discovery of *C9orf72* expansions in FTD and ALS in 2011.^3,4^ Of the 26 individuals with a *C9orf72* expansion who developed subsequent features, 16 did so prior to 2011.

**Table 1:** Sample characteristics

	ALS	ALS + Cog	FTD	FTD + Motor	*P*-value
*n*	152	16	51	22	
Sex (male, %)	91 (59.9)	7 (43.8)	30 (58.8)	12 (54.5)	0.64
Race (%)					0.21
African American or Black	4 (2.6)	2 (12.5)	1 (2.0)	0 (0.0)	
Multi-racial	0 (0.0)	0 (0.0)	1 (2.0)	0 (0.0)	
White	145 (95.4)	14 (87.5)	49 (96.1)	21 (95.5)	
Unknown	3 (2.0)	0 (0.0)	0 (0.0)	1 (4.5)	
Ethnicity (%)					0.66
Latino	3 (2.0)	0 (0.0)	1 (2.0)	0 (0.0)	
Non-Latino	146 (96.1)	15 (93.8)	50 (98.0)	22 (100.0)	
Unknown	3 (2.0)	1 (6.2)	0 (0.0)	0 (0.0)	
Age at death [years, mean (SD)]	63.64 (10.50)	65.76 (9.91)	69.50 (10.85)^a,d^	59.33 (7.79)	<0.001
Age at initial clinical syndrome symptom onset [years, mean (SD)]	59.39 (10.87)	62.25 (10.00)	61.57 (9.22)	54.31 (6.85)^a,b,c^	0.01
Initial clinical syndrome diagnostic delay [months, mean (SD)]	16.94 (18.10)^c,d^	23.33 (31.40)^c^	44.77 (37.02)	41.79 (44.05)	<0.001
Initial clinical syndrome symptom onset to subsequent feature onset [months, mean (SD)]	–	20.09 (19.43)	–	38.13 (45.88)	0.64
Initial clinical syndrome symptom onset to death [months, mean (SD)]	51.00 (49.94)	42.10 (31.62)	95.14 (54.21)^a,b,d^	60.28 (53.17)	<0.001
*C9orf72* expansion present (%)	53 (34.9)	12 (75.0)^a^	21 (41.2)	14 (63.6)	0.002
*C9orf72* expansion absent					0.08
No known genetic aetiology	99 (100.0)	4 (100.0)	27 (90.0)	8 (100.0)	
GBE1	0 (0.0)	0 (0.0)	1 (3.3)	0 (0.0)	
TBK1	0 (0.0)	0 (0.0)	2 (6.7)	0 (0.0)	
FTLD-TDP pathological subtype					0.009*
A	–	–	11 (24.4)	2 (11.1)	
B	–	–	13 (28.9)	13 (72.2)	
C	–	–	15 (33.3)	0 (0.0)	
E	–	–	5 (11.1)	2 (11.1)	
ALS	114 (100.0)	9 (100.0)	–	–	
Not specified	0 (0.0)	0 (0.0)	1 (2.2)	1 (5.6)	
Alzheimer’s disease neuropathologic change					0.15
Not	55 (48.2)	4 (44.4)	19 (42.2)	11 (61.1)	
Low	41 (36.0)	1 (11.1)	20 (44.4)	5 (27.8)	
Intermediate	8 (7.0)	3 (33.3)	2 (4.4)	1 (5.6)	
High	3 (2.6)	0 (0.0)	3 (6.7)	1 (5.6)	
Not available	7 (6.1)	1 (11.1)	1 (2.2)	0 (0.0)	

Demographic, clinical, and genetic data for all individuals, split by initial clinical syndrome (FTD or ALS) and the presence of subsequent motor (FTD + Motor) or cognitive-behavioural (ALS + Cog) features. For continuous variables, mean and standard deviation are provided; *P*-values represent Kruskal–Wallis tests for group comparisons, and differences from aALS, bALS + Cog, cFTD, or dFTD + Motor based on FDR-adjusted significant pairwise Dunn’s tests are indicated. For categorical variables, count and percentage are provided; *P*-values represent *χ*^2^ tests for group comparisons, and differences from aALS, bALS + Cog, cFTD, or dFTD + Motor based on FDR-adjusted significant pairwise *χ*^2^ tests are indicated. * Comparison between FTD and FTD + Motor only.

### Logistic regression

Binary logistic regression revealed increased odds of developing subsequent features in those with *C9orf72* expansions relative to those without (OR = 3.49 [95% CI 1.64–7.80], *P* = 0.002) and decreased odds of developing subsequent features in those with an initial ALS clinical syndrome compared to those with an initial FTD clinical syndrome (OR = 0.25 [95% CI 0.12–0.53], *P* < 0.001). Neither age at initial clinical syndrome onset (OR = 0.98 [95% CI 0.95–1.02], *P* = 0.45) nor sex (OR = 0.65 [95% CI 0.31–1.38], *P* = 0.26) contributed to the odds of developing subsequent features.

### Cox proportional hazards

**Figure 1:**
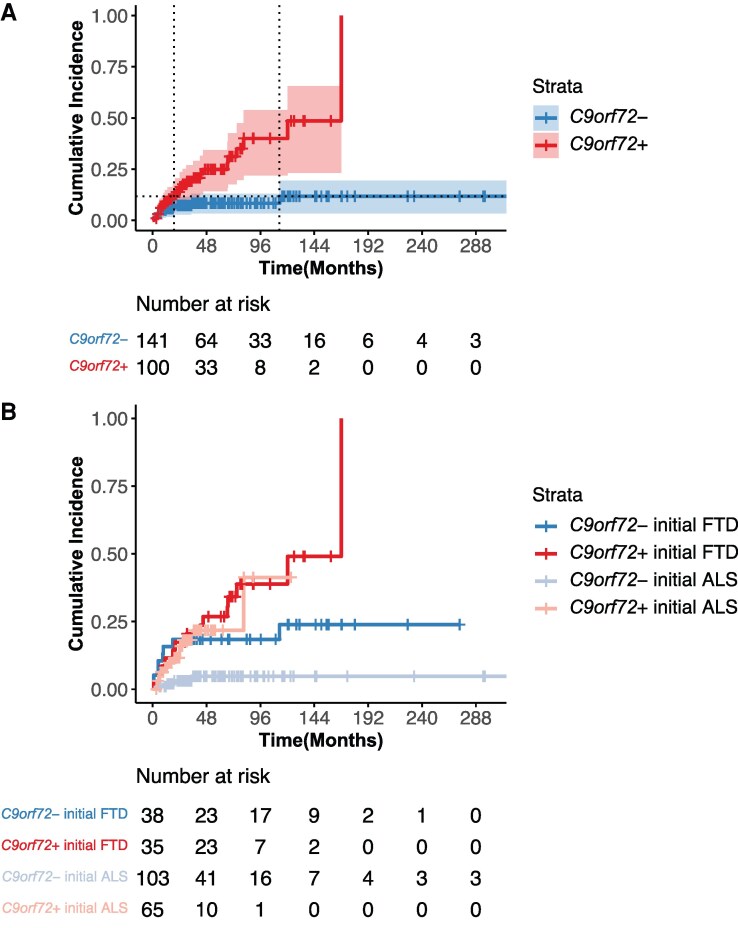
***C9orf72* hexanucleotide repeat expansions increase risk for subsequent feature development.** Kaplan–Meier cumulative incidence plots are displayed stratified by (**A**) the absence (blue, *n* = 141) or presence (red, *n* = 100) of a *C9orf72* expansion alone and (**B**) combined with initial clinical syndrome: initial clinical syndrome of FTD without *C9orf72* expansion (blue, *n* = 38), initial clinical syndrome of FTD with *C9orf72* expansion (red, *n* = 35), initial clinical syndrome of ALS without *C9orf72* expansion (light blue, *n* = 103), and initial clinical syndrome of ALS with *C9orf72* expansion (salmon, *n* = 65). Vertical rises indicate the development of subsequent cognitive-behavioural or motor features. Tick marks indicate censoring due to death. Shading represents 95% confidence intervals. The table shows the number of living individuals in the sample who have not yet developed subsequent features at a given time point. In (**A**) dotted horizontal line represents the highest probability of developing subsequent features reached for a given person without a *C9orf72* expansion in our sample (cumulative incidence 0.12); dotted vertical lines represent the time after symptom onset of the initial clinical syndrome, a given person with (19 months) or without (113 months) a *C9orf72* expansion reaches this probability. Cox proportional hazard analyses, controlling for age at initial clinical syndrome onset and sex, were used to examine the influence of *C9orf72* expansion status, (**A**) alone and (**B**) in combination with initial clinical syndrome, on subsequent feature development. (**A**) Those with *C9orf72* expansions had an increased risk for developing subsequent features compared to those without (HR = 3.78 [95% CI 1.86–7.65], *P* < 0.001). (**B**) In combination with *C9orf72* carrier status, an initial ALS clinical syndrome was associated with a decreased hazard for developing subsequent features (HR = 0.48 [95% CI 0.25–0.95], *P* = 0.03).

Those with *C9orf72* expansions had an increased risk for developing subsequent features compared to those without (HR = 3.78 [95% CI 1.86–7.65], *P* < 0.001). Kaplan–Meier cumulative incidence plots, stratified by *C9orf72* expansion status alone or in combination with initial clinical syndrome, are displayed in Fig. 1. In persons with *C9orf72* expansions, the probability of a given person developing subsequent features reached 0.5 (50%) at 168 months (14 years) after symptom onset of the initial clinical syndrome (Fig. 1). In persons without *C9orf72* expansions, the time at which the probability of a given person developing subsequent features would reach 0.5 is unable to be estimated due to the infrequent development of subsequent features in this group. In combination with *C9orf72* carrier status, an initial ALS clinical syndrome was associated with a decreased hazard for developing subsequent features (HR = 0.48 [95% CI 0.25–0.95], *P* = 0.03). Neither age at initial clinical syndrome onset (HR range across models 0.99–1.00 [95% CI range across models 0.96–1.03]) nor sex (HR range across models 0.74–0.75 [95% CI range across models 0.39–1.43]) was associated with the hazard for developing subsequent features in either model. Tracheostomy was administered to 6 of 241 participants (2.49%), and 6 of 241 participants (2.49%) used permanent assisted ventilation. When we defined survival by death, tracheostomy, or permanent assisted ventilation, we observed similar results to those using death only; individuals with *C9orf72* expansions had an increased risk for developing subsequent features compared to those without (HR = 3.77 [95% CI 1.86–7.65], *P* < 0.001), and, in combination with *C9orf72* carrier status, an initial ALS clinical syndrome was associated with a decreased hazard for developing subsequent features (HR = 0.49 [95% CI 0.25–0.97], *P* = 0.04).

### Regional TDP-43 burden

**Figure 2:**
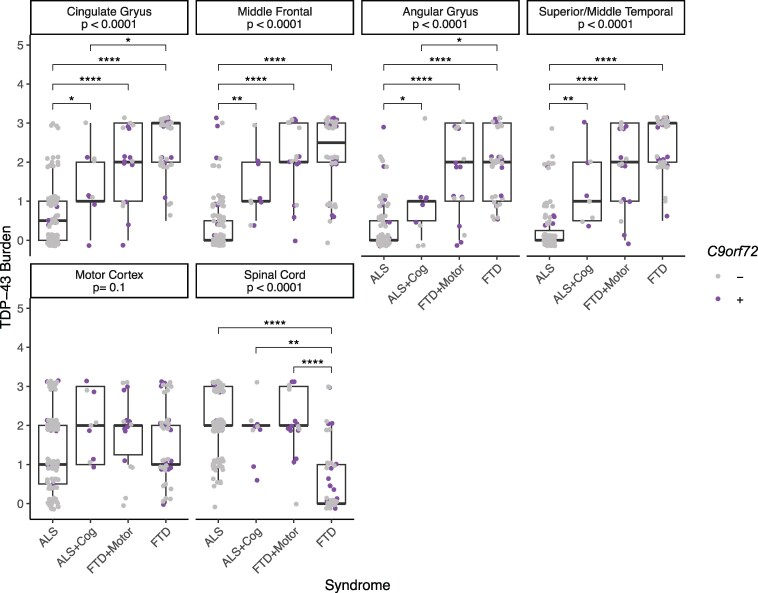
**Regional TDP-43 burden reflects initial clinical syndrome and subsequent features.** The burden of TDP-43 pathology was examined across individuals grouped by initial clinical syndrome and subsequent features. Data points represent the semi-quantitatively scored TDP-43 burden for each individual in characteristic FTD regions (top row: cingulate gyrus (*n* = 175), middle frontal cortex (*n* = 178), angular gyrus (*n* = 175), and superior/middle temporal cortex (*n* = 177)), and characteristic ALS regions [bottom row: motor cortex (*n* = 179) and spinal cord (*n* = 176)]. The FDR-adjusted *P*-values for each Kruskal–Wallis test are displayed by region. Asterisks represent FDR-adjusted significant pairwise Dunn’s tests within region (****P < 0.0001, ***P < 0.001, **P < 0.01, *P < 0.05). Jitter was added around the semi-quantitatively scored TDP-43 burden (0, absent; 0.5, rare; 1, mild; 2, moderate; 3, severe) for individual data point visualization. Individual data points were coloured by the absence (grey) or presence (purple) of a *C9orf72* expansion.

At autopsy, the distribution of TDP-43 pathology across characteristic brain regions reflected both the initial clinical syndrome and subsequent features. First, this was done in characteristic FTD regions, specifically the cingulate gyrus, middle frontal cortex, angular gyrus, and superior/middle temporal cortex (Fig. 2). Pairwise Dunn’s tests revealed a lower burden of TDP-43 pathology in ALS cases without subsequent cognitive-behavioural features compared to FTD cases (with or without subsequent motor features) across all characteristic FTD regions (all FDR-adjusted *P* < 0.001). Further, a lower burden of TDP-43 pathology was observed in ALS cases without subsequent cognitive-behavioural features compared to ALS cases with subsequent cognitive-behavioural features across all characteristic FTD regions (all FDR-adjusted *P* < 0.05). Second, we examined TDP-43 burden in characteristic ALS regions, motor cortex, and spinal cord. In the spinal cord, a lower burden of TDP-43 pathology was observed in FTD cases without subsequent motor features compared to ALS cases (with or without subsequent cognitive-behavioural features) and FTD cases with subsequent motor features (all FDR-adjusted *P* < 0.01). However, no significant differences in TDP-43 burden were observed in the motor cortex across individuals grouped by initial clinical syndrome and subsequent features using the Kruskal–Wallis test (*P* = 0.10).

## Discussion

Individuals with an initial clinical ALS or FTD syndrome with an underlying TDP-43 proteinopathy are at risk for developing subsequent cognitive-behavioural or motor features, respectively, and this risk is heightened for those with disease due to a *C9orf72* hexanucleotide repeat expansion. Regardless of genetic status, there is a modest risk for the development of subsequent features in the first year after symptom onset of the initial clinical syndrome, with 9.0% of persons with *C9orf72* expansions and 5.7% of those without *C9orf72* expansions developing subsequent features in this timeframe. However, by death, 26.0% of those with *C9orf72* expansions develop subsequent features compared to only 8.5% of those without *C9orf72* expansions, in line with previous estimates.^26-28^ While some studies report a median interval of <1 year between the onset of the initial clinical syndrome and subsequent features in those with a *C9orf72* expansion, we observed a median of just under 2 years (22 months).^37^ In our sample, a given person without a *C9orf72* expansion reaches a probability of developing subsequent features of 12% at 113 months after symptom onset of the initial clinical syndrome, after which there is no increase in probability. In contrast, for a given person with a *C9orf72* expansion, that probability is surpassed by 19 months, a 94-month difference. By 168 months (14 years) after symptom onset of the initial clinical syndrome, a person with a *C9orf72* expansion has a 50% probability of developing subsequent features. The mean time between symptom onset of the initial clinical syndrome and death is shorter than 168 months across ALS and FTD groups, and few individuals remain at risk for subsequent feature development at this time point. However, we observed two persons with *C9orf72* expansions who develop subsequent features 10+ years after symptom onset of the initial clinical syndrome, similar to some previously reported intervals.^38^

In this study, we found that the cumulative incidence of subsequent feature development was lower for individuals with an initial ALS clinical syndrome, beyond the effect of *C9orf72* expansion. The time between symptom onset of the initial clinical syndrome and death was shortest for individuals with initial ALS, with or without subsequent feature development, aligning with prior work that found that long-term survivors in the FTD-ALS spectrum were more likely to be individuals with cognitive symptoms at onset.^39^ This difference in survival may partially explain the lower cognitive-behavioural feature development we observed in ALS, although there was no statistical difference in survival between ALS and FTD with subsequent motor features. It is also possible that it is easier for cognitive neurologists to reliably observe motor features in the context of FTD than it is for neuromuscular neurologists to detect cognitive-behavioural features in the context of ALS.

The presence of subsequent feature development was associated with a shorter interval between symptom onset of the initial clinical syndrome and death for both initial FTD and ALS, aligning with previous research,^7-10^ although only individuals with initial FTD without subsequent motor features lived significantly longer than other groups in our sample. We also observed a younger age at onset of cognitive-behavioural symptoms for individuals with initial FTD who develop subsequent motor features (54.31 [6.85]) than those who never develop subsequent features (61.57 [9.22], Table 1).

At autopsy, the distribution of TDP-43 pathology in characteristic regions reflected both the initial syndrome and subsequent features, with relatively preserved spinal cord only in FTD cases without subsequent motor features and relatively preserved neocortical regions only in ALS cases without subsequent cognitive-behavioural features. These data align with previous work highlighting that the distribution and severity of TDP-43 pathology relate to the clinical expression of dementia and motor impairments across the spectrum of TDP-43 proteinopathies^40-42^ and provide important cross-validation for our clinical designations of ALS, FTD, and subsequent features. As expected, we did not observe any occurrences of FTD with subsequent motor features in FTLD-TDP type C.

Future studies are necessary to determine a biological explanation for why individuals with a *C9orf72* expansion have a higher risk for developing subsequent features relative to those without despite sharing common underlying TDP-43 pathology. More people without *C9orf72* expansions than those with them may ultimately develop subsequent features, despite subsequent feature development being a lower probability event, as most individuals with ALS and FTD do not have a *C9orf72* expansion, and studies are needed to elucidate risk factors in this population.^43-45^

As with all epidemiological studies, there are several caveats to consider in our approach. In order to ensure TDP-43 pathology in this study, we relied on neuropathological confirmation, which necessitated a retrospective evaluation of cases. One should consider any potential sources of bias introduced by our primary focus on individuals who participated in brain donation, who tend to be more educated and non-representative of the population per se. Prospective collection of both reported feature onset as well as detailed electrophysiological support and neuropsychological testing would strengthen the study; however, our post-mortem data support our clinical groupings (Fig. 2). It is also necessary for future studies to address other potential modifiers of risk for concomitant cognitive-behavioural and motor impairment such as cognitive and motor reserve factors^46^ and other environmental^47^ and genetic^48,49^ modifiers.

These findings should encourage clinicians to have elevated vigilance for the onset of subsequent cognitive-behavioural and motor features in patients with a *C9orf72* expansion, regardless of initial clinical syndrome, and may warrant dual referrals between cognitive and neuromuscular clinics. Early work likely failed to observe an association between subsequent features and survival in ALS due to a focus on memory rather than FTD-like cognitive/behavioural impairment.^12^ Therefore, it is critical to properly evaluate these patients. This information may also be helpful for patients and families in terms of prognosis and understanding the implications of a *C9orf72* expansion in their family.

## Supplementary Material

fcaf405_Supplementary_Data
