# Supplementary material for: Cumulative incidence of motor and cognitive features in the amyotrophic lateral sclerosis—frontotemporal degeneration spectrum
Source: Brain Commun. 2025 Oct 15;7(6):fcaf405. doi: 10.1093/braincomms/fcaf405 (PMC12596572; doi:10.1093/braincomms/fcaf405)
Supplement: fcaf405_Supplementary_Data [file fcaf405_supplementary_data.docx]

# Supplementary material

**Supplementary Table 1** Onset of subsequent features for each individual, split by initial clinical presentation.

| Subsequent motor features in individuals with FTD | |
| --- | --- |
| 1 | Began walking with irregular gait, several falls, and left foot dropping |
| 2 | Twitching in arms and left eyelid, motor strength was significantly weaker in both hands at follow up |
| 3 | Walking demonstrated mild slowing, some fasciculations, coughed during eating |
| 4 | Dysarthria, difficulty drinking through a straw, stumbles though no falls, weakness in hands and lost dexterity |
| 5 | Began losing agility and dexterity, slow in movement, began walking with a slow, shuffling gait, falls, incontinence, and difficulty handling a fork |
| 6 | Shortness of breath, followed by weakness in hands |
| 7 | Difficulty swallowing and dysarthria |
| 8 | Slurred speech, swallowing difficulty, and possible tongue fasciculations |
| 9 | Speaking less, marked trouble with verbal output, gravelly voice, occasional problem swallowing |
| 10 | Began to fall and had some difficultly with right leg stiffness and difficulty clearing the right foot from the floor |
| 11 | Weakness in right arm followed by problems swallowing |
| 12 | Complained of speech difficulty and gradually progressed to involve the left hand and left leg |
| 13 | Noted some muscle wasting at visit followed by fasciculations in the upper extremities bilaterally, weakness in hands and legs, and slowing gait |
| 14 | Multiple falls, progressive weakness beginning with left leg, followed by right hand and right leg, difficulty walking and climbing steps |
| 15 | Dysarthria which worsened, falls, and hand weakness |
| 16 | Trouble eating, takes a long time to chew food, may be some coughing while eating, followed by progressive loss of use of arms and legs |
| 17 | Walks very, very slowly, dragging feet, and trips and falls, difficulty using utensils, choking on liquids, losing weight |
| 18 | Dysphagia, fasciculations of tongue and in the limbs, hyperreflexia, a mildly spastic gait, and nasally speech |
| 19 | Slurring speech, gait imbalance, and upper extremity weakness |
| 20 | Weight loss, progressive hand weakness, difficultly walking, falls, and fasciculations |
| 21 | Progressive speech difficulties and dysphagia |
| 22 | Chart unavailable for re-review |

| Subsequent cognitive-behavioral features in individuals with ALS | |
| --- | --- |
| 1 | Very prominent personality change, apathetic - laissez-faire; short term memory less sharp -repeats self |
| 2 | Progressive behavioral and language changes, sentences were progressively shorter until only able to communicate basic needs with short sentences |
| 3 | Grammatical errors and progressive difficulty with written and oral communication |
| 4 | Developed odd behaviors including giving away large sums of money to telemarketers, spending thousands on publishers clearing house sweepstakes, and donating large sums of money, collecting thousands of CDs from TV ads, ordering numerous tools and socks, and became ritualistic about timing of sleep and meals |
| 5 | Noted individual appears to have frontal lobe dysfunction |
| 6 | Repetition of stories, difficulty with multitasking and attention, inappropriate comments, impulse control issues, somewhat lacking in empathy and at times is agitated |
| 7 | Change in behavior, repetitively climbing steps and pacing, obsessively locking doors and windows |
| 8 | Impaired working memory and judgement, slow at times to follow concepts, cognitive issues described as consisted with FTD |
| 9 | Poor insight, didn’t recall ALS diagnosis discussion, impaired working memory and judgment, some difficulty following commands |
| 10 | Partner had cognitive concerns, some problems on cognitive testing, change in personality, obsessive writing and pacing and TV watching |
| 11 | Cognitive dysfunction noted with poor ECAS and behavioral change |
| 12 | Executive and social difficulties, reduced initiation and motivation, poor judgement and has been wanting to give away money to everyone, limited insight, poor empathy |
| 13 | Cognitive impairment on exam |
| 14 | Poor ECAS performance |
| 15 | Difficulty understanding abstract information and techniques/strategies, decreased insight into safety with functional tasks, denies difficulty with tasks |
| 16 | Chart unavailable for re-review |

**Supplementary Figure 1** Representative images of semi-quantitatively scored TDP-43 burden (**A**: 0, absent; **B**: 0.5, rare; **C**: 1, mild; **D**: 2, moderate; **E**: 3, severe) in cervical spinal cord. All scale bars represent 50μm.
